# Value of willingness to pay for a QALY gained in Iran; a modified chained-approach

**DOI:** 10.1186/s12913-021-07344-w

**Published:** 2021-12-14

**Authors:** Seyedeh-Fariba Jahanbin, Hasan Yusefzadeh, Bahram Nabilou, Cyrus Alinia

**Affiliations:** Department of Health Management and Economics, School of Public Health, Urmia University of Medical Sciences, Urmia, Iran

**Keywords:** Willingness to Pay, QALY, Chained-Approach, Cost-effectiveness threshold, Iran

## Abstract

**Background:**

Due to the lack of a constant Willingness to Pay per one additional Quality Adjusted Life Years gained based on the preferences of Iran’s general public, the cost-effectiveness of health system interventions is unclear and making it challenging to apply economic evaluation to health resources priority setting.

**Methods:**

We have measured this cost-effectiveness threshold with the participation of 2854 individuals from five provinces, each representing an income quintile, using a modified Time Trade-Off-based Chained-Approach. In this online-based empirical survey, to extract the health utility value, participants were randomly assigned to one of two green (21121) and yellow (22222) health scenarios designed based on the earlier validated EQ-5D-3L questionnaire.

**Results:**

Across the two health state versions, mean values for one QALY gain (rounded) ranged from $6740-$7400 and $6480-$7120, respectively, for aggregate and trimmed models, which are equivalent to 1.35-1.18 times of the GDP per capita. Log-linear Multivariate OLS regression analysis confirmed that respondents were more likely to pay if their income, disutility, and education level were higher than their counterparts.

**Conclusions:**

In the health system of Iran, any intervention that is with the incremental cost-effectiveness ratio, equal to and less than 7402.12 USD, will be considered cost-effective.

## Background

How to decide on the allocation of limited resources among many health care interventions determines the efficiency of a given health system [1]. Over the past three decades, developed countries have been well-used of value-based medicine tools, especially economic evaluation, to prioritize healthcare [2]. Economic evaluation is a general term for a set of techniques used to identify, measure, and evaluate the costs and consequences of health interventions. Both the inputs and the outputs of one intervention are compared to other competing interventions for decision making [3]. One form of economic evaluation, cost-utility analysis, which presents results in terms of cost per Quality-Adjusted Life Years (QALY), is well suited for health policy-making [4]. The QALY is a two-dimensional measure, combining both longevity and quality of health. Quality of life, known as health utility, is generally expressed on a numerical scale ranging from 0 (represents death) to 1 (state of perfect health) [5]. In this technique, interventions with an incremental cost-effectiveness ratio (ICER) less than the social value of Willingness to Pay (WTP) for a QALY gained will be considered cost-effective [6].

The value of WTP per QALY, known as the cost-effectiveness threshold, has been measured in developed countries through cross-sectional empirical surveys and is updating over time [7]. These studies are highly heterogeneous in terms of sample size, level of study, type of sample, extraction tools of health utility values, scenarios depicting health status with varying degrees of severity, and overall study planning [8–10]. Hence, their results have different validity and generalizability, and they produce very mixed results even for the same populations [10].

For those countries where this value has not been empirically measured, the World Health Organization (WHO) has recommended thresholds of one to three times the gross domestic product (GDP) per capita [11]. Although resource allocation techniques have developed in developing countries like Iran, misallocation of resources is still high, and the social welfare loss due to this distortion is considerable [12–14].

In this regard, few efforts have been made in Iran to extract the value of WTP per QALY gained by Lankarani et al. [15] and Moradi et al. [16–19]. But, due to the low sample size, sample selection bias, methodological deficits, and disregarding the population and income diversities, they have failed to produce results representing the general population’s actual preferences. Three studies by Moradi et al. have estimated the monetary value of a QALY among patients with heart and diabetes diseases. Less than 200 patients in one or two hospitals were studied who could not be disaggregated for population and income subgroups. Therefore, their results cannot be generalized to public health [16, 18, 19]. In population-based studies by Moradi et al. [17] and Lankarani et al. [15]. One thousand two healthy individuals in Tehran and 651 healthy individuals in Shiraz were investigated respectively. These studies applied the direct approach to estimate the financial value of one QALY, which does not have the accuracy and generalizability of the Chained method [20, 21].

They have estimated the value of WTP per QALY gained to range from 0.23 to 0.95 times of GDP per capita, which, compared with the amount of Iranians ability to pay, appears to be very low and irrational. Therefore, they cannot be an appropriate benchmark for judging the cost-effectiveness of different interventions in the health system. To fill this knowledge gap, we estimated the social value of WTP per QALY gained by modifying the constraints listed above.

## Methods

The methodology of this paper is taken from the study of Robinson et al. [20] which uses a Chain Approach. We conducted this study in 4 steps; introduction of Chained Approach, questionnaire design, sampling and data collection, and data analysis.

## Introduction of chained approach

To derive a cost-effectiveness threshold, which means the maximum monetary value of one year of full quality, we have developed a Chain Approach using a two-step process. In the first stage, we extracted the health utility, and in the next step, the maximum amount that the Iranian population is willing to pay for each definitive additional QALY is estimated. The Chain Approach provides one or more pre-designed health scenarios that are hypothetically described and thus extract potentially different individuals’ utility and preferences for the same health status. These health conditions were defined and developed using the validated EQ-5D-3L questionnaire for the Iranian population [20, 22, 23]. The Chain Approach is understandable, applicable, less susceptible to bias, highly sensitive to diagnostic differences in health status, and consistent. For this reason, the inclusion criteria are less restricted, and almost everyone in the community can participate [20]. Another strength of this approach is that its results have higher social generalizability than the direct approach [22]. It is crucial for achieving the objectives of the present study because otherwise, they would not be appropriate for policy-making. Not using the direct approach in this regard was for its two significant limitations.

One of the Chain Approach functions is to standardize the value of the QALY index, as it yields the same QALY value for all studied participants. Therefore, this exercise considers different life traded times for different utility values so that the multiplying of these two values gives the same QALY for all the subjects. We considered the 0.1 QALY gained as an equal valuation baseline for all the hypothetical scenarios. Every 0.1 QALY is equivalent to 36.5 days of full quality of life.

In the “direct method”, participants are asked to rate their utility value for a pre-defined health status plotted on a health thermometer numbered between 0 (equivalent to death) to 100 (equivalent to full health). Then applying Time Trade-Off (TTO) tool, a customized time for each expressed utility value are considered to define the avoidance of one QALY loss. Finally, respondents are directly asked for their maximum WTP to prevent the loss of this one QALY. For example, if a person rates a disutility of 0.25 (0.5) for a specific health condition, mainly described in the EQ-5D questionnaire, they are asked to express the maximum amount of WTP to avoid living with the condition for four (two) years [21]. Studies have shown that the direct method, due to the consideration of large health losses, causes WTP to exceed the ability of individuals to pay. This problem is solved in the Chained Approach by considering the minor health losses that people are accustomed to paying for [21, 23–25].

### Questionnaire design

**Fig. 1:**
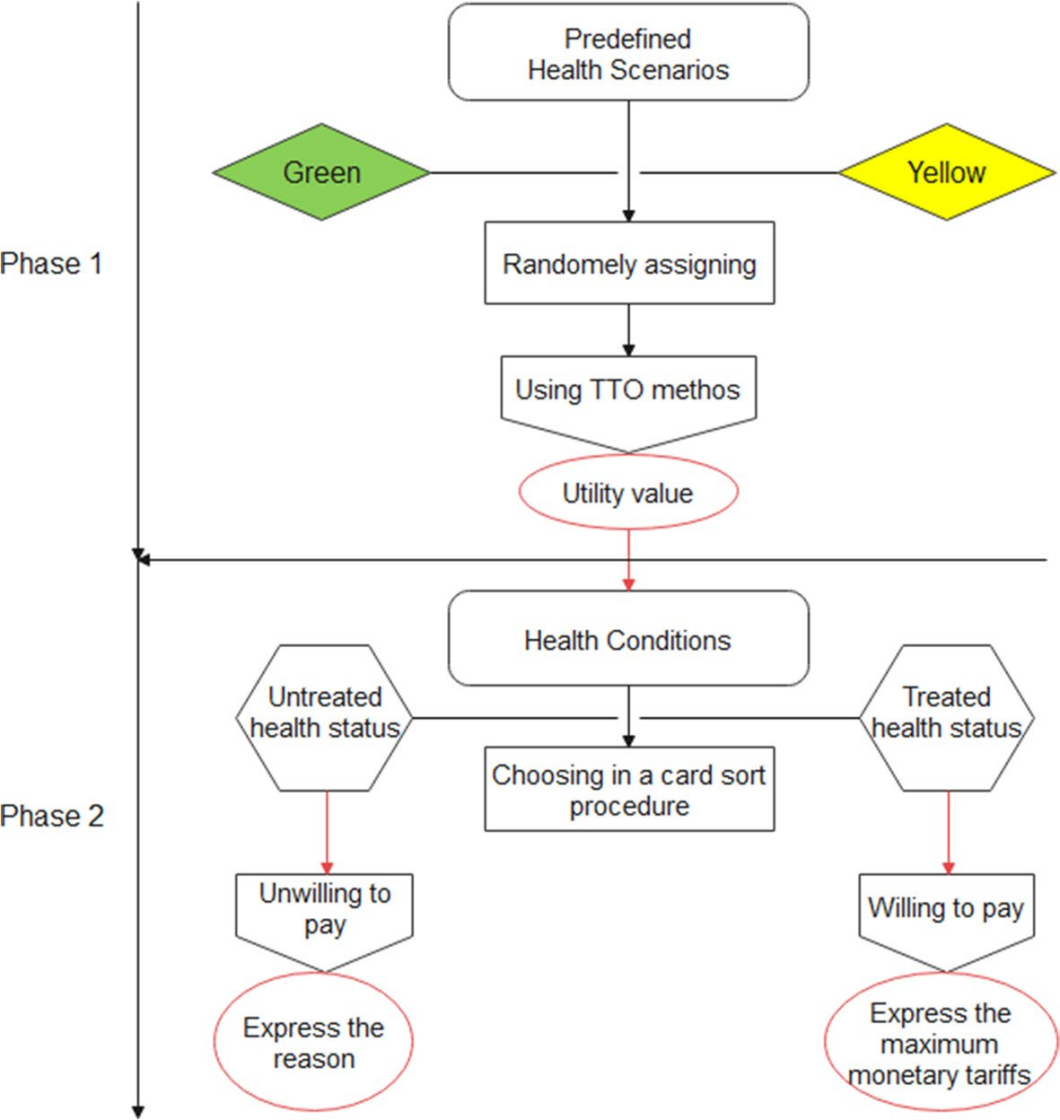
Flowchart of elicitation the health states utilities and WTP per a QALY gained

The data collection tool was a web-based, flexible, and in Persian questionnaire. In addition to demographic information (age, gender, height, weight, education level, marital status, employment status, living place, household dimension, monthly household expenses, and health insurance status), this questionnaire consists of two parts of extraction of health utility and elicitation of WTP value per a QALY gained as depicted in Fig. 1.

### Health scenarios and utilities

In the second part of the questionnaire, we defined two hypothetical health conditions of Green and Yellow health state scenarios. These health scenarios were determined based on five dimensions of health in the European Quality of Life Five Dimension Five Level Scale (EQ-5D-3L) questionnaire, including mobility, self-care, usual activities, pain/discomfort, and anxiety/depression These dimensions are defined in three levels of no problems, some problems, and extreme problems. The highest and lowest health utility values were for individuals to choose options 1 (11111) and 3 (33333), respectively, for all five dimensions. Based on Robinson et al. recommendation [20], the Green and Yellow health scenarios, which we predetermined with the health statuses of 21121 (having some problems in mobility, some pain/discomfort, no problems in self-care, usual activities, and anxiety/depression) and 22222 (having some pain/discomfort and some problems in mobility, self-care, usual activities, and anxiety/depression), respectively, are defined as follows, and we ask the participants to indicate their health utility for them.

After randomly assigning each of the two presumed health scenarios to the study participants, we sought to derive their health utility using the TTO method. We first asked them about their age, and according to the Iranian life table, we calculated the specific age-sex standardized life expectancy for each person. Life expectancy at birth is reported by the latest WHO report for the Iranian population at an average of 75.77 years. It was 74.5 and 76.6 years for males and females, respectively. The expected remaining age in this study is rounded up in a monthly unit.

**Fig. 2:**
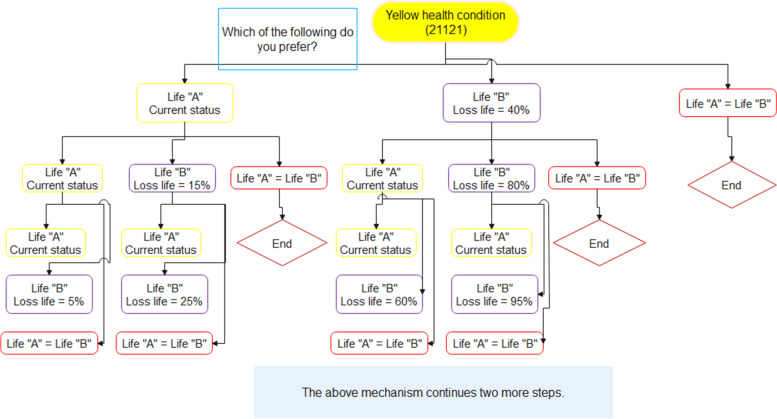
Flowchart of eliciting procedure of utility value

We explained two lives, “A” and “B” to the respondents for each of the scenarios. In life “A”, the individual spends all rest of his/her life (e.g., 20 years) with the assumed health status (Green/Yellow), but in life “B” spends part of this life (e.g., 12 years) with complete health status without any health problems, but loses the rest completely. We asked the participants which of the three options of “Life A”, “Life B”, and “there is no difference between life A and life B you choose. Initially, in all questions, 60% of the remaining years in “life B” can be lived with full health quality (e.g., 12 out of 20). By selecting any of the options, except option three, they entered the next step, which again the same three options are exposed to their selection. The difference is that if they had chosen the “Life A” (“Life B”) option, the percentage of life with full health quality in the “Life B” option has increased (decreased). In general, this process of extracting utility continues in a maximum of five steps, and a total of 95 final answers are possible, including two extreme values. If an individual had chosen the “Life A” option at all stages, he/she would eventually be asked that you are willing to lose a few weeks or days of their remaining life in exchange for being in perfect health condition for the rest of their life. Also, if a respondent had chosen only the “Life B” option at all steps, he/she would eventually be asked the additional question of whether you are willing to lose your entire life to avoid the presumed health condition. To derive the participants’ utility preferences, we have continued the questions to the point that they knew the value of lives A and B alike (Fig. 2). Using the formula below, we estimated the health utility value of each participant.

$$Health\ utility=1-\frac{Lost\ life\ time\ \left( in\ month\right)}{Standard\ remained\ life\ time\ \left( in\ month\right)}$$

### Elicitation of WTP per a QALY gained

After determining of disutility value (is equal to 1 - utility value), we have estimated the WTP value per QALY gained for each participant. For this purpose, after introducing the willingness to pay questions, the participants were asked how much money they were willing to pay to eliminate the presumed health status (Green/Yellow) they would be living with at a given period. To measure this goal, we formulated and described two health conditions with and without treatment. In untreated status, the individual will spend a certain amount of time in the assumed health scenario (Green/Yellow health status) and spend the rest of their lives in perfect health. This specified early-life time is obtained by dividing 0.1 QALY by the extracted health disutility of each participant and presented to his/her by month. For example, the length of time considered for a hypothesized health status with a 0.2 (0.4) utility was six months (three months), resulting in a loss of 0.1 QALY. In explaining this situation, participants were urged to ignore any financial loss and focus only on reducing their health-related quality of life in the presumed health conditions. In treated status, participants are in perfect health condition for the rest of their lives without any problem and suffering.

In the following, in a card sort procedure, we asked each participant that is willing to pay any amount to receive such treatment. If the answer was yes, the person had moved on to the next step; otherwise, they will be asked to choose one of the following six pre-determined reasons for their decision. 1) The risk of health is very low, and it does not pose a particular concern, 2) However, I will get full health, so it is not worth paying, 3) This treatment is valuable, and it should be paid for, but the government must make the payment, 4) This treatment is valuable and worth paying, but I cannot pay for it, 5) This situation is not so bad, I can live with it, and finally, 6) For other reasons I refuse to pay. But for the respondents who were willing to pay, the monetary tariffs were randomly given to them, which varied depending on the respondents’ answers in the successive bids. It means that if people were willing to pay the initial amount, the next question (s) would indicate a 20 percent higher amount; otherwise, the amount asked would be 20 percent lower. There are three options for answering each of these questions; I will definitely pay, I will definitely not pay, and I am not sure if I will pay this amount.

**Fig. 3:**
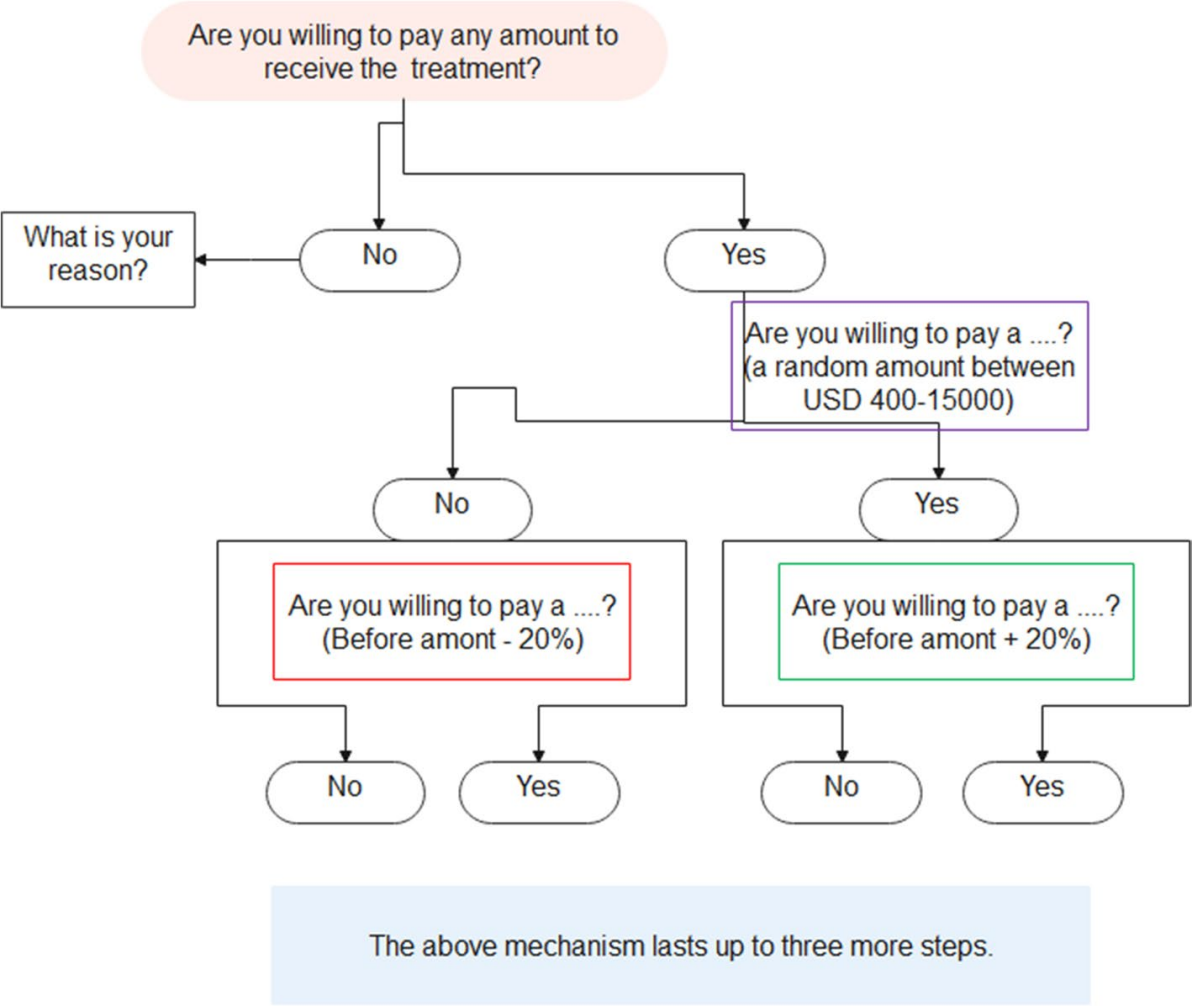
Flowchart of general procedure of eliciting of WTP per QALY

This process continues for a maximum of five bids so that participants eventually choose option three, and we set the last amount they will definitely pay for treatment as their WTP to avoid 0.1 QALY, and we multiplied the result by 10 to get WTP per whole QALY for each person. The minimum and maximum bids were USD 400 and USD 15000, respectively. The respondents who had opted for “I will definitely pay” in all bids and have reached a maximum of $ 15000 have been asked the additional question of whether they are willing to pay more to receive the proposed treatment. Of course, the figures beyond the [Q3 + 1.5 * (Q3-Q1)] were considered outliers, and their effect has been considered in the calculations. We finally asked the participants if they were willing to pay a small amount for the defined treatment method, in case they had chosen the “I will definitely not pay” option at all bids (Fig. 3).

To assess the validity of the questionnaire, we examined its questions in two stages by health economics and policymakers as well as by 50 participants in the study. If necessary, these questions were corrected, completed, clarified, and finalized. These potential corrections have been made to improve understanding of questions, correct writing and grammatical errors, optimize questionnaire completion times, and other potential issues. Besides, to assess the reliability, the modified questionnaire was completed by these 50 individuals in the following two weeks again. The concordance of the results was evaluated using the test-retest test.

### Sampling and data collection

This online-based cross-sectional survey includes a representative sample of the Iranian population in terms of gender and age groups, randomly selected from five provinces of Tehran, East Azerbaijan, West Azerbaijan, Kohgiluyeh and Boyer-Ahmad, and Kurdistan. These provinces respectively represent income quintiles in Iran. The country’s last census results in 2019 show that the average household income was USD 4832.28. The provinces of Tehran and West Azerbaijan had the highest ($ 7115.78) and lowest ($ 2941.23) average household incomes, which were in the first and fifth income quintiles, respectively. Also, Kohgiluyeh and Boyer-Ahmad Provinces, East Azerbaijan, and Kurdistan were third to fifth quintiles with USD 4899.04, USD 4109.98, and USD 3615.18, respectively [26]. The minimum number of samples needed in each of these provinces, representing the country’s income quintiles, was considered 506 individuals aged 18-65 years. Having reading and writing skills and having no specific (thalassemia, dialysis, kidney transplant, multiple sclerosis, and hemophilia) or incurable illnesses have been the other inclusion criteria.

### Data analysis

Since in the second part of the study, the WTP value was extracted for each 0.1 QALY, multiplying the result by ten is obtained the WTP value per QALY gained at the individual level. We calculated and presented the trimmed and untrimmed results using mean (standard deviation and 95% confidence interval) and median (interquartile range) WTP for each QALY gained for all age-sex subgroups and also for both the Green and Yellow health scenarios. According to the boxplot definition, outliers are data that are larger than the result of the Q3+1.5*(Q3-Q1), provided that this value is not more than the 99th percentile [27]. In this relation, Q1 and Q3 are the first and third quartiles, respectively. By plotting the histogram of the maximum stated WTP by participants, we assured about its normal distribution. Therefore, for the statistical comparison of two groups and more than two independent groups at an error level of 0.01, we used parametric tests of one-way ANOVA and Independent t-test, respectively. Log-linear Multivariate OLS regression was used to examine the factors affecting the WTP per QALY gained, in which we defined the WTP logarithm as the dependent variable.

## Results

The participation rate in this study was 96.37%. As of 3386 people, 3263 have expressed their desire to participate in the study, and among them, 409 (12.07%) did not meet the inclusion criteria, so 2854 (84.29%) were studied. One thousand five hundred twenty-five and One thousand three hundred twenty-nine respondents were randomly assigned to the Green and Yellow health scenarios, respectively. As shown in Table 1, all socioeconomic characteristics of the study participants were statistically equally distributed between the two scenarios, except for gender and being household head factors. As in the Yellow health scenario, the share of men and household heads was higher than in another scenario.

**Table 1:** Sample characteristics

Socio-economic variables	Green scenario (21121)	Yellow scenario (22222)	***P***-value
N	1525	1329	
Mean age (SD)	34.79 (11.16)	34.17 (10.84)	0.13
Education years (SD)	15.58 (4.32)	15.45 (4.23)	0.45
Household dimension (SD)	4.08 (1.68)	4.09 (1.65)	0.87
Household monthly expenditure (SD)	265.24 (200.19)	267.47 (155.82)	0.74
Female (%)	861 (56.46)	636 (47.86)	< 0.01
Employed (%)	819 (53.70)	706 (53.12)	0.76
Urban (%)	1,401 (91.87)	1,230 (92.55)	0.50
Having health insurance (%)	1367 (89.64)	1187 (89.32)	0.78
Married (%)	797 (52.26)	657 (49.44)	0.10
Being head of household (%)	482 (31.61)	473 (35.59%)	0.03

*Abbreviations*: *SD* Standard deviation

We have seen that the age-gender distribution of the respondents is very similar to that of the general population (Table 2).

**Table 2:** Relative distribution of Iranian population and study sample by age and gender

Age groups	Male		Female	
	Population	Sample	Population	Sample
18-24	20.99%	13.41%	20.70	24.18%
25-54	69.08%	77.08%	68.74%	70.74%
+ 55	9.93%	9.51%	10.56%	5.08%

The difference in mean sex-adjusted life expectancy was not significant between the two groups. This value was obtained for males in the range of 41.82-42.70 years and females in 48.04-48.83 years. However, the median age of women in the Yellow health scenario was significantly greater than their counterparts in the Green health scenario.

Table 3 reports central tendency statistics (mean and median values) of aggregate and three different scenarios of trimmed (aggregate without outliers, protesters, and both) values of WTP per QALY estimates and its variations by the health state scenarios. Across the two health state versions, mean values for one QALY gain (rounded) ranged from $6740 to $7400 for the aggregate model. These values in trimmed models ran from $6485 to $7750 for green health state and $7120 to $8240 for yellow health state. The lowest and highest mean WTP per QALY were related to models without outliers and protesters, respectively. The mean value for the Yellow health state was approximately 10% higher than the mean for the Green health state similarly in both models. This relative difference in medians across the two health states was about 9 and 12% for aggregate and trimmed models, respectively. After trimming the outlier values in each health state, means of WTP per QALY estimates and health utility were respectively reduced by 4 and 3.4% relative to the base case. Table 3 also reports the statistics on non-traders, protesters, and outliers. The number of non-traders in the Yellow health scenario is about 5% more than in another scenario. While, the number of protesters and outliers in the Green health scenario was reported to be 29 and 25% more than the rival scenario, respectively. Of the 335 participants who were unwilling to pay the minimum amount for treatment, 71 (21.19%) people said that However, I would get full health, so it is not worth paying, 68 (20.30%) cases stated their reason for not having money despite the value of the treatment, 55 (16.42%) participants believed that the risk of health is very low and it does not pose a particular problem, 50 (14.93%) individuals believed that this situation is not so bad and I can live with it, 43 (12.84%) participants said that the treatment is valuable, but the government must pay for it, and finally, 48 (14.33%) individuals mentioned other reasons.

**Table 3:** Aggregate and trimmed values of WTP per QALY estimates^*^ in $US by the health state scenarios

Values	Green health state (21121) ***N***= 1525 (53.43%)	Yellow health state (22222) ***N***= 1329 (46.57%)	Total ***N***= 2854 (100.00%)
**Model 1.** Aggregate WTP per a QALY			
Mean (SD)	6737.90 (2282.57)	7402.12 (2347.78)	7047.20 (2329.05)
95% CI	[6573.02 - 6902.78]	[7221.97 - 7582.27]	[6925.02 - 7169.39]
Median (IQR)	6694.90 (2666.67)	7287.12 (4444.44)	6737.90 (3666.67)
Mean/GDP per capita	1.23	1.35	1.28
Median/GDP per capita	1.22	1.33	1.23
Health utility (SD)	0.545 (0.35)	0.461 (0.33)	0.506 (0.35)
Non-traders^a^ [N(%)]	178 (11.67)	178 (13.39)	356 (12.47)
Protesters^b^ [N(%)]	200 (13.11)	135 (10.15)	335 (11.74)
Outliers^c^ [N(%)]	211 (13.84)	147 (11.06)	358 (12.54)
**Model 2.** Aggregate WTP per a QALY without protesters			
Mean (SD)	7754.52 (2834.44)	8238.31 (2564.76)	7984.59 (2638.85)
95% CI	[7466.22 -7928.72]	[8095.34 – 8448.40]	[7846.16 – 8123.03]
Median (IQR)	7144.83 (2512.84)	7788.24 (3856.73)	7408.43 (3134.23)
Health utility (SD)	0.561 (0.33)	0.473 (0.32)	0.517 (0.33)
**Model 3.** Aggregate WTP per a QALY without outliers			
Mean (SD)	6484.55 (1938.00)	7122.03 (1978.46)	6799.44 (1988.99)
95% CI	[6334.95 - 6634.16]	[6959.34 - 7284.72]	[6688.32 - 6910.56]
Median (IQR)	6357.90 (1626.79)	7122.03 (2122.23)	6737.90 (2266.667)
Health utility (SD)	0.527 (0.34)	0.446 (0.32)	0.489 (0.34)
**Model 4.** Aggregate WTP per a QALY without protesters and outliers			
Mean (SD)	7648.74 (2185.94)	8121.97 (2163.32)	7925.86 (2318.50)
95% CI	[7326.29 – 7824.53]	[7936.44 – 8307.50]	[7796.34 – 8055.35]
Median (IQR)	6938.66 (1506.74)	8009.53 (2017.45)	7510.87 (1738.22)
Health utility (SD)	0.532 (0.33)	0.483 (0.33)	0.509 (0.33)

*WTP* Willingness to pay, *QALY* Quality Adjusted Life Years, *SD* Standard deviation, *CI* Confidence Interval, *IQR* Interquartile range, *GDP* Gross Domestic Production

^a^ Respondents who traded off no time at all in the TTO technique

^b^ Non-payers

^c^ According to the definition of a boxplot (exceeding Q3 + 1.5 * (Q3-Q1) but lower than 99 percentile)

* in the estimated WTP per QALY ‘non-traders’ and ‘outliers’ are omitted and Iranian GDP per capita was considered as 5494.06 $US in 2017

Table 4 reports the value of sex-adjusted WTP per QALY estimates across different income subgroups by the health state scenarios, in which Income subgroups are classified according to the per capita monthly household expenditure. The findings confirmed expected positive relations between WTP per QALY and income levels for both scenarios, but this relation was statistically significant only among females. The fifth income quintile had an average of 17 and 27% more WTP for gaining one QALY than the first quintile, respectively, in the Green and Yellow health state scenarios.

**Table 4:** Value of sex-adjusted WTP per QALY estimates over income subgroups^*^ in $US for Green and Yellow health state scenarios

Gender	Income subgroups (USD)	Number	Mean (SD)	95% CI	***P***-value
Green health state scenario					
Male	< 110	47	7262.17 (1784.36)	[6464.69 - 8059.64]	0.082
	110-220	198	6221.29 (2049.07)	[5795.81 - 6646.77]	
	220-330	230	6835.48 (2561.56)	[6374.36 - 7296.61]	
	> 330	189	6947.57 (2543.89)	[6441.40 - 7453.73]	
Female	< 110	90	6122.73 (2460.75)	[5406.73 - 6838.72]	0.005
	110-220	266	6355.71 (2237.99)	[5966.04 - 6745.38]	
	220-330	318	7040.42 (2195.48)	[6688.71 - 7392.12]	
	> 330	187	7146.48 (2002.45)	[6715.54 - 7577.41]	
Yellow health state scenario					
Male	< 110	38	6459.55 (1699.96)	[5599.59 - 7319.50]	0.145
	110-220	215	7074.54 (2177.68)	[6649.04 - 7500.04]	
	220-330	243	7458.03 (2370.77)	[7033.48 - 7882.59]	
	> 330	197	7554.21 (2198.81)	[7106.74 - 8001.68]	
Female	< 110	40	6314.02 (2720.07)	[5158.98 - 7469.06]	0.038
	110-220	206	7320.39 (2591.21)	[6829.05 - 7811.74]	
	220-330	239	7539.72 (2421.38)	[7105.13 - 7974.31]	
	> 330	151	7999.27 (2223.98)	[7484.06 - 8514.48]	

*SD* Standard Deviation, *CI* Confidence Interval

* Income subgroups are classified according to the per capital monthly household expenditure

We reported the findings of the regression analysis of WTP values on participant’s characteristics for aggregate and trimmed models in terms of the health scenarios in Table 5. In both models, respondents were more likely to pay if their income, disutility, and education level were higher, and this association was found for single and not being a household head respondents only in the aggregate model. We observed a higher propensity to pay amongst employed respondents in aggregate Green health state and respondents with a higher Body Mass Index in trimmed Yellow health state.

**Table 5:** OLS regression analysis to examine the factors affecting WTP per QALY values

	Dependent variable: Ln(willingness to pay)					
Variables	Aggregate model			Trimmed model^a^		
	Green health state	Yellow health state	Total	Green health state	Yellow health state	Total
Ln(life expectancy)	-0.103 (0.18)	-0.296^*^ (0.18)	-0.121 (0.13)	0.017 (0.14)	-0.251 (0.14)	-0.122 (0.09)
Ln(BMI)	0.519^*^ (0.27)	0.466^*^ (0.26)	0.115 (0.19)	0.194 (0.20)	-0.425^**^ (0.16)	-0.088 (0.11)
Ln(education years)	0.543^***^ (0.12)	0.194^*^ (0.11)	0.373^***^ (0.08)	0.247^***^ (0.07)	0.158^**^ (0.07)	0.229^***^ (0.06)
Ln(per capita monthly household expenditure)	0.249^***^ (0.06)	0.342^***^ (0.06)	0.288^***^ (0.04)	0.102^**^ (0.04)	0.116^***^ (0.04)	0.112^***^ (0.03)
Female = 1	-0.082 (0.10)	0.085 (0.08)	0.077 (0.06)	-0.020 (0.06)	0.008 (0.06)	0.014 (0.05)
Employed = 1	0.299^***^ (0.09)	0.048 (0.09)	0.151^**^ (0.06)	-0.126^*^ (0.05)	-0.026 (0.06)	-0.080 (0.05)
Married = 1	-0.103 (0.09)	-0.221^**^ (0.09)	-0.184^***^ (0.06)	-0.003 (0.05)	-0.045^**^ (0.05)	-0.020 (0.04)
Urban = 1	0.162 (0.14)	0.185 (0.14)	0.169^*^ (0.10)	0.109 (0.08)	0.249 (0.09)	0.178^**^ (0.07)
Having basic insurance = 1	0.080 (0.13)	0.149 (0.13)	0.136 (0.09)	0.073 (0.07)	0.197 (0.09)	0.124^*^ (0.07)
Head of household = 1	-0.374^***^ (0.12)	-0.114 (0.12)	-0.272^***^ (0.09)	-0.166^*^ (0.09)	-0.085 (0.09)	0.131^*^ (0.07)
Health utility	-0.265^**^ (0.11)	-0.118^**^ (0.12)	-0.213^***^ (0.08)	-0.234^**^ (0.10)	-0.194^**^ (0.11)	-0.221^***^ (0.09)
Constant	5.368 (1.24)	9.430 (1.19)	6.641 (0.84)	6.628 (0.98)	9.130 (0.96)	7.778 (0.56)
Observations	1525	1329	2854	1314	1182	2496
R-squared	0.17	0.16	0.15	0.21	0.20	0.17
Prob > F	< 0.001	< 0.001	< 0.001	<0.001	0.003	<0.001

Robust standard deviation in parentheses

*** *p*<0.01, ** *p*<0.05, * *p*<0.1

^a^ Outliers are trimmed according to the definition of a boxplot (exceeding Q3 + 1.5 * (Q3-Q1))

## Discussion

Although Low and Middle-income countries (LMICs) face severe resource constraints, they neglected economic evaluation tools for improving healthcare resources efficiency. Therefore, a few empirical studies have been conducted in these countries, including Iran, to extract the value of WTP per QALY from a societal viewpoint. All of them, except for Thailand’s study [28], due to the small sample size, non-generalizable to the general public, and methodological limitations, has failed to provide statistically acceptable figures for healthcare policymaking. This large cross-sectional population-based study aimed to estimate a constant social monetary value for a QALY by applying TTO-based Chained-Approach, which we designed to overcome limitations of contingent valuation method used by Moradi et al. [16–19] and Lankarani et al. [15]. Except for considering some predetermined random values of WTP per a QALY with a limited range of 400-15000 USD and over for those who selected the upper limit in the card sort procedure to avoiding a starting and ceiling points biases, and also controlling the stated outlier WTP, two major features of our Chained Approach were keeping ‘small’ and ‘constant’ QALY gains, 0.1 QALY, for all elicited different health utility values across the respondents. These features resulted in high validity of the results and minimized the possibility of anchoring bias.

Although the median and mean values of WTP per QALY obtained in each health scenario were statistically significantly different, this difference was unexpectedly and substantially less than findings of other similar studies. Of course, when we omitted the outlier values from the calculations, this difference was no longer significant, and when we excluded the ‘protest’ zero bids from the analysis, a more considerable distance was observed. This proximity of the mean and mean values may be due to the lower shares of an outlier, protestors, and non-traders figures compared to other similar studies [15, 20, 23]. Since Robinson et al. [20] have shown, direct and those versions of Chained methods used by EuroVaQ project [25], Baker et al. [29], and Pinto Prades et al. [30] have obtained extremely high and unacceptable mean WTP per a QALY, but It is not clear to us that the characteristics of the chained method have led to such an important finding which highlights the need for another comparative study.

The WTP per QALY values obtained at the Individual data level for women and the aggregate level showed a positive and significant association with the household expenditure level, which is a proxy for the household’s ability to pay. This relationship is not confirmed for men at the micro-level, that it is suggested their reasons be examined in a study. Provincial results show that the mean (SD) of WTP values for the provinces of Tehran, Kohgiluyeh and Boyer-Ahmad, East Azerbaijan, Kurdistan, and West Azerbaijan were equal to 8520.33 (2743.62), 7744.82 (2566.32), 7085.75 (2398.74), 6248.56 (2284.53), and 5656.41 (2221.57) respectively which had a 95% correlation with the household ability to pay.

As expected, the health utility value related to the Green scenario was statistically significantly higher than in the Yellow health scenario in general and also in all age-sex subgroups. This result confirmed the overall consistency of the participants. Findings show that Iranians are willing to pay an average of 6200-7900 USD (558- 711 million Rial) to obtain a definite extra QALY in different age-sex subgroups, which we did not see a statistically significant difference between them. However, this value was affected by the health state scenarios since the mean WTP per a QALY gained in the Yellow health scenario being statistically significantly higher than the Green health scenario (7402.12 versus $ 6737.90) and are as much as 1.23 and 1.35 times greater than Iranian GDP per capita, respectively. This result represents a plausible range and is very close to Iranians’ ability to pay. The obtained WTP value per QALY also had a significant positive linear relationship with their income, especially in women. The estimated WTP values are about three times higher than those found by non-representing disease-based Moradi et al. models [16, 17, 19] and also population-based Lankarani et al. [15] approach. In the study of Lankarani et al. [15] the contribution of Zero WTP in various hypothetical health scenarios varied between 23 and 66%, which could negatively affect the final results, and perhaps this explains why the achieved gap between the mean and median of estimated WTP in all of these scenarios is dramatically high.

**Fig. 4:**
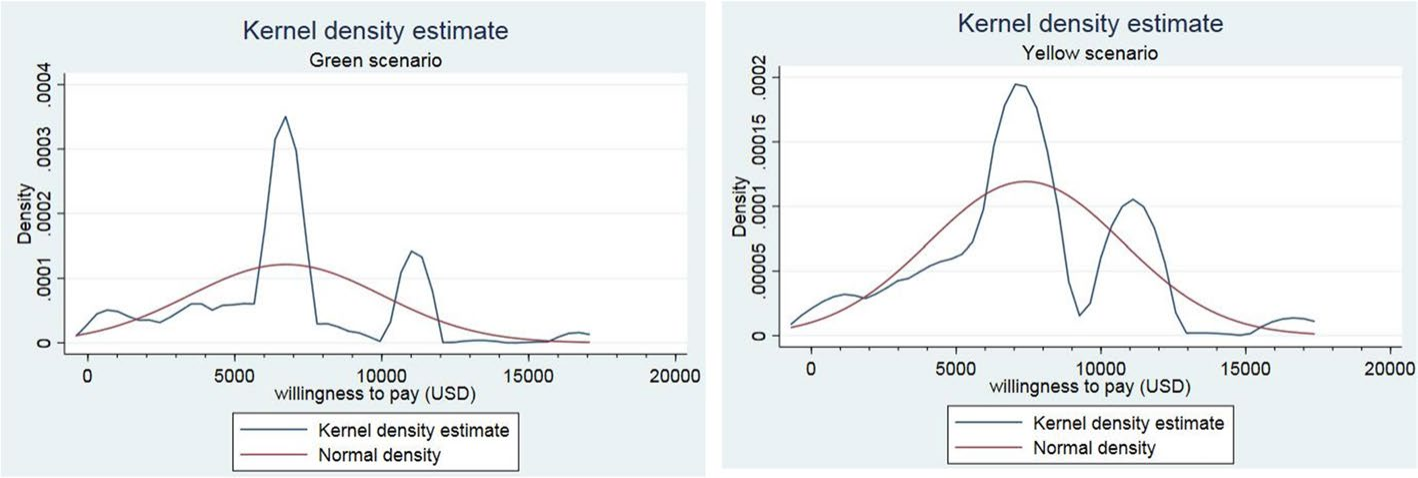
Distribution of WTP per a QALY gained by health state scenarios

Surprisingly, kernel density figures show that despite existing significant outliers, WTP values per QALY gained had a normal distribution in both the Green and Yellow scenarios. The results have shown evidence supporting this claim, as the mean and median values are very close, even in subgroups analysis. But it is seen that the variation and kurtosis of the distribution of the WTP per QALY gained in the Yellow health scenario is much lower than in the Green health scenario, which could be evidence of its higher validity (Fig. 4). Therefore, it can be claimed that the results are valid and efficient, which the researchers believe was the result of the large sample size and also the application of the modified Chained Approach methodology in this study. Since the achieved average values of WTP per a QALY gained is very close to the one GDP per capita figure, the WHO’s recommended cost-effectiveness threshold at the spectrum of one-to-three times GDP per capita is accepted, but we suggest this range be changed to one-to-two times GDP per capita. Because as Nimdet et al. [9] showed in a systematic review study, except for few studies, in all countries, the estimated average of WTP per a QALY gained was less than two times GDP per capita.

What factors statistically determine the WTP values to obtain a definite QALY depends on the sample size, the assumed health scenario and existing outlier data in the study. Although, in all analytical models, the log-linear multivariate regression analysis demonstrates the expected significant positive relationship between WTP values and per capita income and education level, exactly like what was found in Pennington et al. [21] and Lankarani et al. [15] studies.

Even though we performed the sample collection as an online survey, the respondents’ relative distribution of age and sex subgroups was very close to the general public and allowed for many samples to participate in the study (Table 2). However, this study had clear limitations, such as considered only one constant QALY, 0.1 QALY, for all respondents and used the TTO format as the sole method of extracting health utility. Therefore, we cannot make any judgments about the sensitivity of achieved WTP values to scale effect. In addition, although participants reported an average higher utility value for the green health scenario, they reported a statistically significantly lower monetary value to prevent the loss of a QALY than the yellow health scenario. However, the median values of WTP per QALY obtained for these health conditions are close to each other, as it is estimated at USD 7287.12 for the yellow health condition and USD 6694.90 for the green health condition. According to the theory, the WTP per QALY should be equal in different scenarios, but the findings led to a lack of approval of the scope sensitivity of our approach and raised this hypothesis that respondents may find it better to lose 0.1 QALYs due to a less severe health state for a longer time.

Besides, the high generalizability due to considering population and income diversity, large sample size, and participation of the general public in the study has led it to have undeniable advantages over many other similar studies. We modified Robinson’s Chained Approach model by taking age-sex-standard life expectancy into account. We think not using the population life table in the TTO approach in other studies has led to underestimating standard remained life time and health utility values, leading to a high mean WTP per QALY. For example, the Iranian remained life years in the age group of 50-55 are 30.54 and 27.34 years, respectively, according to the age-standardized life expectancy and expectancy at birth, which can significantly affect calculating the health utility values. Therefore, due to an error in calculating a QALY, the result of WTP per QALY could be biased.

Also, given that we have extracted individual ex-post WTP per QALY, according to the findings of Bibinac et al. [31], the results could be underestimated. This is because the participants face limited ability to pay when they need treatment and exclusion of risk aversion. However, these restrictions do not exist in the ex-ante frame, which leads to a more accurate estimation.

Applying the results of this study for health resource allocation in an efficient way requires removing the practical barriers and constraints that exist in the Iranian health system. Firstly, most health decision-makers do not have the proper knowledge about optimal prioritizing of healthcare interventions based on cost-effectiveness analysis. Secondly, there are no explicit resource allocation rules and guidelines that raise serious conflicts of interest, as key Iranian healthcare decision-makers are mostly themselves providers of tertiary care services. Finally, the absence of a native protocol for conducting economic evaluation studies makes it impossible to compare the results of different studies with each other, and the amount of WTP presented in this research.

## Conclusion

We have measured the willingness to pay for a QALY gained with the participation of 2854 individuals from five provinces using modified Time Trade-Off-based Chained-Approach. In this online-based empirical survey, to extract the health utility value, participants were randomly assigned to one of two green (21121) and yellow (22222) health scenarios designed based on the earlier validated EQ-5D-3L questionnaire by Goudarzi et al. [32]. Across the two health state versions, mean values for one QALY gain ranged from $6740-$7400 and $6480-$7120, respectively, for aggregate and trimmed models, equivalent to 1.35-1.18 times of the GDP per capita.

## Data Availability

The datasets generated and/or analyzed during the current study are not publicly available for confidentiality reasons since individual privacy could be compromised but are available from the corresponding author on reasonable request.

## References

[1] Angelis A, Kanavos P, Montibeller G (2017). Resource allocation and priority setting in health care: a multi-criteria decision analysis problem of value?. Glob Policy.

[2] Drummond MF, Sculpher MJ, Claxton K, Stoddart GL, Torrance GW (2015). Methods for the economic evaluation of health care programmes.

[3] Brazier J, Ratcliffe J, Saloman J, Tsuchiya A (2016). Measuring and valuing health benefits for economic evaluation.

[4] Edwards RT, Winrow E. Cost–utility analysis of public health interventions. Appl Health Econ Public Health Pract Res. 2019;177-8.

[5] MacKillop E, Sheard S (2018). Quantifying life: understanding the history of quality-adjusted life-years (QALYs). Soc Sci Med.

[6] Bertram MY, Lauer JA, De Joncheere K, Edejer T, Hutubessy R, Kieny M-P, Hill SR (2016). Cost–effectiveness thresholds: pros and cons. Bull World Health Organ.

[7] Revill P, Ochalek J, Lomas J, Nakamura R, Woods B, Rollinger A, Suhrcke M, Sculpher M, Claxton K. Cost-effectiveness thresholds: guiding health care spending for population health improvement. A report by the Centre for Health Economics, University of York, for the International Decision Support Initiative (iDSI) Centre for Health Economics, University of York 2015.

[8] Claxton K, Martin S, Soares M, Rice N, Spackman E, Hinde S, Devlin N, Smith PC, Sculpher M. Systematic review of the literature on the cost-effectiveness threshold. In: Methods for the estimation of the National Institute for Health and Care Excellence cost-effectiveness threshold. edn.: NIHR Journals Library; 2015. 10.3310/hta19140 PMC4781395 25692211

[9] Nimdet K, Chaiyakunapruk N, Vichansavakul K, Ngorsuraches S (2015). A systematic review of studies eliciting willingness-to-pay per quality-adjusted life year: does it justify CE threshold?. PLoS One..

[10] Ryen L, Svensson M (2015). The willingness to pay for a quality adjusted life year: a review of the empirical literature. Health Econ.

[11] Robinson LA, Hammitt JK, Chang AY, Resch S (2017). Understanding and improving the one and three times GDP per capita cost-effectiveness thresholds. Health Policy Plan.

[12] Powell-Jackson T, Mills A (2007). A review of health resource tracking in developing countries. Health Policy Plan.

[13] Kapiriri L, Norheim OF, Heggenhougen K (2003). Using burden of disease information for health planning in developing countries: the experience from Uganda. Soc Sci Med.

[14] Youngkong S, Kapiriri L, Baltussen R (2009). Setting priorities for health interventions in developing countries: a review of empirical studies. Tropical Med Int Health.

[15] Lankarani KB, Ghahramani S, Moradi N, Shahraki HR, Lotfi F, Honarvar B (2018). Willingness-to-pay for one quality-adjusted life-year: a population-based study from Iran. Appl Health Econ Health Pol.

[16] Moradi N, Rashidian A, Mohammadi T, Alamdari S, Oliaei Manesh A (2015). Willingness to pay for one quality adjusted life years in patients with diabetes. Value Health.

[17] Moradi N, Rashidian A, Nosratnejad S, Olyaeemanesh A, Zanganeh M, Zarei L (2019). Willingness to pay for one quality-adjusted life year in Iran. Cost Eff Resour Alloc.

[18] Moradi N, Rashidian A, Nosratnejad S, Olyaeemanesh A, Zanganeh M, Zarei L (2019). The worth of a quality-adjusted life-year in patients with diabetes: an investigation study using a willingness-to-pay method. PharmacoEconomics-Open.

[19] Moradi N, Rashidian A, Rasekh HR, Olyaeemanesh A, Foroughi M, Mohammadi T (2017). Monetary value of quality-adjusted life years (QALY) among patients with cardiovascular disease: a willingness to pay study (WTP). Iran J Pharm Res.

[20] Robinson A, Gyrd-Hansen D, Bacon P, Baker R, Pennington M, Donaldson C, Team E (2013). Estimating a WTP-based value of a QALY: the ‘chained’approach. Soc Sci Med.

[21] Pennington M, Baker R, Brouwer W, Mason H, Hansen DG, Robinson A, Donaldson C, Team E (2015). Comparing WTP values of different types of QALY gain elicited from the general public. Health Econ.

[22] Asim O, Petrou S (2005). Valuing a QALY: review of current controversies. Exp Rev Pharmacoecon Outcomes Res.

[23] Olofsson S, Gerdtham U-G, Hultkrantz L, Persson U (2019). Value of a QALY and VSI estimated with the chained approach. Eur J Health Econ.

[24] Ahlert M, Breyer F, Schwettmann L (2013). What you ask is what you get: willingness-to-pay for a QALY in Germany.

[25] Donaldson C, Baker R, Mason H, Pennington M, Bell S, Lancsar E, Jones-Lee M, Wildman J, Robinson A, Bacon P. European value of a quality adjusted life year. Final publishable report 2010.

[26] Ahmadnia E, Kharaghani R, Maleki A, Avazeh A, Mazloomzadeh S, Sedaghatpisheh T, Jalilvand A, Molae B (2016). Prevalence and associated factors of genital and sexually transmitted infections in married women of Iran. Oman Med J.

[27] Olofsson S, Gerdtham U-G, Hultkrantz L, Persson U. Chained approach vs contingent valuation for estimating the value of risk reduction. Department of Economics, Lund University Working Papers 2016, 2016(34).

[28] Thavorncharoensap M, Teerawattananon Y, Natanant S, Kulpeng W, Yothasamut J, Werayingyong P (2013). Estimating the willingness to pay for a quality-adjusted life year in Thailand: does the context of health gain matter?. ClinicoEconomics Outcomes Res.

[29] Baker R, Bateman I, Donaldson C, Jones-Lee M, Lancsar E, Loomes G, Mason H, Odejar M, Prades J-LP, Robinson A (2010). Weighting and valuing quality-adjusted life-years using stated preference methods: preliminary results from the Social Value of a QALY Project. Health Technol Assess.

[30] Pinto-Prades JL, Loomes G, Brey R (2009). Trying to estimate a monetary value for the QALY. J Health Econ.

[31] Bobinac A, van Exel J, Rutten FF, Brouwer WB (2014). The value of a QALY: individual willingness to pay for health gains under risk. Pharmacoeconomics.

[32] Goudarzi R, Sari AA, Zeraati H, Rashidian A, Mohammad K, Amini S (2019). Valuation of quality weights for EuroQol 5-dimensional health states with the time trade-off method in the capital of Iran. Value Health Reg Issues.

